# A cell transportation solution that preserves live circulating tumor cells in patient blood samples

**DOI:** 10.1186/s12885-016-2330-1

**Published:** 2016-05-06

**Authors:** Steingrimur Stefansson, Daniel L. Adams, William B. Ershler, Huyen Le, David H. Ho

**Affiliations:** HeMemics Biotechnologies Inc., 12111 Parklawn Drive, Rockville, MD 20852 USA; Creatv MicroTech, Inc., 1 Deer Park Dr., Monmouth Junction, NJ 08852 USA; Institute for Advanced Studies in Aging (IASIA), 6400 Arlington Blvd. Suite 940, Falls Church, VA 22042 USA; Nauah Solutions, LLC., 1616 Anderson Rd., McLean, VA 22101 USA

**Keywords:** Circulating tumor cells, CTC preservation, Transportation, Live CTC

## Abstract

**Background:**

Circulating tumor cells (CTCs) are typically collected into CellSave fixative tubes, which kills the cells, but preserves their morphology. Currently, the clinical utility of CTCs is mostly limited to their enumeration. More detailed investigation of CTC biology can be performed on live cells, but obtaining live CTCs is technically challenging, requiring blood collection into biocompatible solutions and rapid isolation which limits transportation options. To overcome the instability of CTCs, we formulated a sugar based cell transportation solution (SBTS) that stabilizes cell viability at ambient temperature. In this study we examined the long term viability of human cancer cell lines, primary cells and CTCs in human blood samples in the SBTS for transportation purposes.

**Methods:**

Four cell lines, 5 primary human cells and purified human PBMCs were tested to determine the viability of cells stored in the transportation solution at ambient temperature for up to 7 days. We then demonstrated viability of MCF-7 cells spiked into normal blood with SBTS and stored for up to 7 days. A pilot study was then run on blood samples from 3 patients with metastatic malignancies stored with or without SBTS for 6 days. CTCs were then purified by Ficoll separation/microfilter isolation and identified using CTC markers. Cell viability was assessed using trypan blue or CellTracker™ live cell stain.

**Results:**

Our results suggest that primary/immortalized cell lines stored in SBTS remain ~90 % viable for > 72 h. Further, MCF-7 cells spiked into whole blood remain viable when stored with SBTS for up to 7 days. Finally, live CTCs were isolated from cancer patient blood samples kept in SBTS at ambient temperature for 6 days. No CTCs were isolated from blood samples stored without SBTS.

**Conclusions:**

In this proof of principle pilot study we show that viability of cell lines is preserved for days using SBTS. Further, this solution can be used to store patient derived blood samples for eventual isolation of viable CTCs after days of storage. Therefore, we suggest an effective and economical transportation of cancer patient blood samples containing live CTCs can be achieved.

## Background

The primary mechanism of metastatic spread begins with dissemination, or shedding, of cancerous epithelial cells from tumor sites into the circulation. These circulating tumor cells (CTCs) travel throughout the body, adhere to organ vascular beds, infiltrate the tissue, grow and impair organ function [1–3].

Animal studies have shown that organ colonization of injected tumor cells is very efficient, i.e. ~80 % of injected tumor cells extravasate into organs [4–7]. However, the majority of those extravasated cells do not form tumors, thus their metastatic potential through CTC dissemination is, in most cases, very inefficient. Despite ~10^6^ tumor cells are being shed into the circulation per gram of tumor tissue every 24 h, less than 1 % of shed CTCs remain alive 24 h after dissemination [8–11]. This loss of CTC viability has been attributed to many factors including fragility, shear stresses in the circulation, anoikis and destruction by the immune system [12–16]. Interestingly, studies imply that the injected cancer cells that form primary tumors have different biological properties than their progeny populating the metastatic tumors [17, 18].

Analyzing human CTCs is technically challenging because of their extreme rarity in cancer patient blood samples (commonly ≤ 10 CTCs among 10^9^ total blood cells), their inherent heterogeneity and instability [16, 19–21]. CellSearch® is the only FDA approved and clinically validated CTC assay that isolates CTCs, used as a prognostic indicator of survival for breast, prostate, and colorectal cancer patients. This system was developed based on affinity-based isolation procedures and biomarker presence [22–25]. The CellSearch® CTC Test requires blood samples to be collected in CellSave tubes which contain a fixative solution that allows storage of blood samples for up to 3 days, but also kills the CTCs. The need for CTC fixation is necessitated because of their inherent fragility [19–21, 26, 27], but besides killing the CTCs, fixation also crosslinks extra- and intracellular biomolecules that can compromise some molecular analysis [28, 29].

Examining live CTCs has the potential of advancing the knowledge of cancer metastasis by interrogating the underlying biological activity of cells which cannot be accomplished on dead cells, i.e. mRNA profiling, culturing, etc. [28–34]. While many devices and techniques have been developed to capture live CTCs from patient blood samples, the blood sample is usually processed on-site and within hours of collection to retain viability [30, 35–41]. Therefore, transportation of live CTCs in blood samples between research institutions is often not possible due to the high rate of CTC senescence in whole blood.

We first examined the ability of the SBTS to preserve viability in primary cells, cell lines and cancer cells spiked into blood cells at ambient temperature for storage/transportation purposes. We then extended our studies, examining the effect of the SBTS on patient blood samples with live CTCs. Our data suggest that cells can be stored for days without harming the viability of the cells and that CTCs in patient blood samples can also be stored for 6 days at room temperature and retain viability. While further studies on the potential use of the SBTS to preserve live CTCs in transporting blood samples is needed, we suggest that cells can be kept viable when stored long term in blood at ambient temperatures.

## Methods

### Antibodies and reagents

The sugar based cell transportation solution (SBTS) is a non-toxic proprietary mixture of high and low molecular weight carbohydrates (HemSol™). CellSieve™ CTC micro-filtration system from Creatv Microtech (Rockville, MD) using a low-pressure vacuum system which isolates CTCs based on size exclusion, > 7 micron, as previously described [42–44]. Stains used on the CTCs were CellTracker™ Blue CMAC live cell stain (Thermo-Fisher). FITC labeled Pan-cytokeratin clone C11 (Sigma), which recognizes human cytokeratins 4, 5, 6, 8, 10, 13 and 18. Phycoerythrin (PE) labeled anti-human epithelial cell adhesion molecule (EpCAM), clone 1B7 (eBioscience). Alexa Fluor® 594 labeled anti-human CD45, clone 2D1 (Novus) and DRAQ5™ fluorescent DNA probe (Thermo-Fisher). Exposure times (and ex/em wavelengths of the Leica microscope filters), respectively, were: Blue CMAC: 35 msec, (350 nm/460 nm); FITC-CK, 500 msec (470 nm/525 nm); PE-EpCAM, 500 msec (546 nm/585 nm); Alexa Fluor594 500 msec (594 nm/645 nm); . DRAQ5 600 msec (640 nm/690 nm). Samples were analyzed using a Leica fluorescent microscope and imaged with a Leica camera and Leica Microsystems imaging software.

### Cell lines, primary cells and blood samples

Chinese hamster ovary cells (CHO), human embryonic kidney cells (HEK 293), human umbilical vein endothelial cells (HUVEC) and human epithelial colorectal adenocarcinoma cells (CACO-2) human breast cancer epithelial cells (MCF-7), in addition to primary human cells were purchased from ATCC. Peripheral blood mononuclear cells (PBMCs) were isolated from whole blood samples obtained from healthy volunteers using Ficoll separation (GE Healthcare) as described by the manufacturer. Healthy volunteer blood samples were procured with signed informed consent and IRB approval by Western IRB. Patient blood samples were collected with signed informed consent and an IRB with Inova Fairfax Hospital.

### Incubation of cells and cell lines with HemSol™ transportation solution

HemSol™ preservation experiments with primary human hepatocytes, B-Cells, kidney cells, mesenchymal stem cells and non-small cell (NSC) lung carcinoma were performed in collaborations with AscentGene Inc. (Gaithersburg, MD). Approximately 10^5^–10^7^ cells were kept in their respective growth media prior to treatment with HemSol™. Live cells were enumerated by trypan blue exclusion. Cells were then mixed with concentrated HemSol™ (2-6X) for a final concentration of 1X HemSol™. The cells were then stored in the solution for the indicated time at ambient temperature, after which the HemSol™ was washed away using the respective cell media and live cells determined using trypan blue exclusion and noted in Table 1.

**Table 1:** Viability of cells kept in SBTS for the indicated time^a^ at ambient temperature. Cells (10^5^-10^7^), were mixed with SBTS and cell viability was assessed using trypan-blue exclusion after washing and resuspending cells in their respective serum containing media^b^

Cell Type	Cell Condition	Time in SBTS (Days)^a^	% Live Cells^b^
CHO	Suspension	4	95–98
CHO	Adherent	3	95–99
HEK 293	Adherent	3	92–98
HUVEC	Suspension	3	90–98
HUVEC	Adherent	3	93–96
CACO-2	Adherent	3	92–98
Primary hepatocytes	Adherent	7	85–92
PBMC	Suspension	3	> 95
B-Cells	Suspension	4	95–98
Primary kidney cells	Suspension	7	85–90
Mesenchymal stem cells	Suspension	9	92–98
NSC lung carcinoma	Suspension	3	80–92

Live cells were determined from at least 3 experiments

### Analysis of MCF-7 cells spiked into normal whole blood with HemSol™ transportation solution

Human breast cancer cell line (MCF-7) was labeled with CellTracker™ Green (Invitrogen) according to manufacturer’s protocols and cells were then washed to remove remaining free dye. CellTracker™ Green is an intracellular stain that only labels live cells and is retained by cell progeny, allowing for multigenerational tracking. Approximately 10,000 of the labeled MCF-7 cells were added to 8 ml of normal whole blood. 4 ml of that blood sample was then mixed with HemSol™ and 4 ml was incubated without the HemSol™ at RT for 4 days. After the incubation, the blood was filtered and the filters put into tissue culture with DMEM, 10 % serum at 37 °C, 5 % CO_2_ and imaged after 3 days in culture. A second set of labeled MCF-7 cells were kept in whole blood for 7 days at RT followed by filtration using the CellSieve™ CTC micro-filtration system from Creatv Microtech, as previously described [42–44] and imaged.

### Analysis of cancer patient blood samples incubated with HemSol™ transportation solution

To determine the preservation of live CTCs in HemSol™, duplicate whole blood samples (7.5 ml) from 1 breast cancer patient, 1 pancreatic cancer patient and 1 lung cancer patient were collected into EDTA vacutainers and the contents of one tube immediately transferred to a 15 ml conical tube containing 6X concentrated HemSol™, which was diluted to 1X HemSol™ with the blood and mixed for complete dispersal. The duplicate blood sample was transferred to a conical tube containing PBS at the same volume as the concentrated HemSol™, and mixed similarly. The blood samples were kept for 6 days at RT without agitation and then fractionated by mixing the samples 1:1 with PBS and overlaying the samples onto Ficoll. The samples were centrifuged at 400 × g for 30 min at 18 °C in 50-mL centrifuge tubes according to manufacturer’s instructions. HemSol™ did not interfere with the density separation of red blood cells from the PBMC buffy coat layer. The collected buffy coat layer was pelleted by centrifugation and resuspended in 1 ml PBS with CellTracker™ Blue CMAC Dye cell live stain (Invitrogen) for 45 min at RT according to manufacturer’s instructions. After incubation, unbound dye was removed by centrifugation and resuspension of the cell pellet in 2 ml PBS followed by re-centrifugation. The resulting cell pellet was then resuspended in PBS containing 1 % paraformaldehyde (PFA), 5 mM EDTA and incubated for 20 min at RT. After fixation, the cells were filtered using CellSieve™ micro-filtration system (Creatv Microtech) [42–45] set at a flow rate of 5 ml/min. The captured cells were then permeabilized with PBS containing 0.4 % Triton X-100 in PBS, washed twice with 4 ml PBS and stained as previouly described [42–45].

## Results and discussion

Analyzing physical parameters of cancer patient blood samples are routine procedures performed in clinical labs around the world. A recent addition to the arsenal of clinical blood testing procedures in oncology practices is the enumeration of circulating tumor cells (CTCs). These are cancerous cells of epithelial origin that are shed from solid tumors and found in the circulation of many cancer patients which can be used as indicators of patient survival. However, CTCs are fragile cells that die and disintegrate rapidly in whole blood samples [16, 19–21]. Therefore, enumeration of CTCs requires patient blood collection into specialized tubes containing fixatives that preserve CTC morphology, but sacrifice viability. The blood samples collected into fixatives keep cell integrity for at least 3 days, allowing them to be transported to other labs for enumeration and further analysis. However, many biological analyses are compromised as cells are dead and imbued with fixative [28, 29]. Keeping CTCs alive in whole blood samples long enough for timely and economical transportation is a challenge as whole blood is an exceptionally harsh media for non-hematopoietic cells.

The SBTS is a proprietary mixture of simple and complex carbohydrates that was initially formulated to stabilize human red blood cell (RBC) structure and antigen properties upon desiccation for use in reference labs [46]. Oxygen binding properties of desiccated RBCs and platelet structure and function stored at 4 °C for up to 2 weeks are also protected in the presence of these carbohydrates [47, 48]. Those results prompted us to examine whether the SBTS could also stabilize the structure of live nucleated cells. Table 1 shows stabilization of live cell kept in SBTS for 3–9 days at RT.

These data show that immortalized, and primary cells both adherent and in suspension are kept live in SBTS for at least 3 days at ambient temperature. Of special notice is that anchorage dependent cells, including human umbilical vein endothelial cells (HUVEC), human primary kidney cells, Chinese hamster ovary cells (CHO) and human non-small cell (NSC) lung carcinoma cells remain viable in suspension for at least 3 days in SBTS. The positive results of these studies prompted us to examine whether SBTS can preserve cells in other inhospitable media, such as whole blood. Many CTCs are of epithelial origin, which means that they are generally anchorage dependent and undergo apoptosis when in suspension.

Before analyzing CTCs from patient blood samples, we first performed experiments with live MCF-7 epithelial breast cancer cells, which are also anchorage dependent, spiked into whole blood as described [42]. CellTracker™ Green pre-labeled MCF-7 cells were spiked into 4 ml of normal whole blood with or without SBTS. This CellTracker™ dye in a non-fluorescent cell permeable esterase substrate that becomes fluorescent after cleavage by intracellular esterases. The cleavage traps the fluorescent dye inside the cells and it is retained even after cell divisions, however the dye is lost when the integrity of the cell is compromised.

**Fig. 1:**
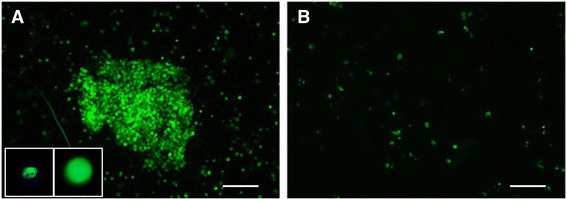
MCF-7 cells were labeled with CellTracker™ live dye and spiked into whole blood either in the presence or absence of SBTS. After 4 days of storage at RT, the blood sample was filtered and the filters put into tissue culture media (DMEM +10 % FBS). After 3 days in tissue culture, MCF-7 cells were dividing and forming colonies (Panel **a**). Insets in panel A shows higher resolutions of an MCF-7 cell stained with DAPI (*left*) and CellTracker™ live dye (*right*) (box = 35 μm). MCF-7 cells that were incubated in whole blood for the same period did not grow (Panel **b**). Bar equals 100 μm

After 4 days in whole blood at RT, the MCF-7 cells were isolated using CellSieve™ microfilters and the filters were put into cell culture media and allowed to grow for 3 days. Figure 1a shows that MCF-7 cells spiked into whole peripheral blood treated with SBTS were able to proliferate and formed colonies post-isolation. In contrast, MCF-7 cells isolated from whole blood in the absence of SBTS had far fewer viable cells and none of the cells proliferated, even after 7 days in culture (Fig. 1b). Interestingly, in the absence of SBTS, live MCF-7 cells isolated from blood did not proliferate.

**Fig. 2:**
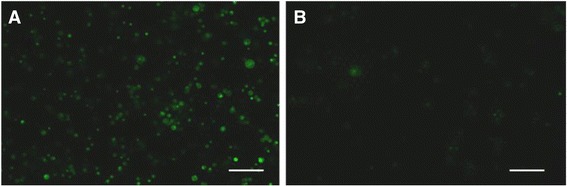
MCF-7 cells were labeled with CellTracker™ live dye and spiked into whole blood either in the presence or absence of SBTS. After 7 days of storage at RT, the blood samples were imaged. The samples incubated with SBTS shows live MCF-7 cells (Panel **a**). In comparison, the absence of SBTS, very few live MCF-7 cells were observed (Panel **b**). Bar equals 100 μm

Additionally, live MCF-7 cells were further kept in normal whole blood for 7 days at RT followed by filtration and imaging. Figure 2a shows live MCF-7 cells in the SBTS treated blood sample with very few live MCF-7 cells observed in whole blood in the absence of SBTS (Fig. 2b).

We then proceeded to analyze whether the SBTS was able to keep CTCs alive in cancer patient blood samples. Live CTCs are fragile cells that generally need to be isolated rapidly from blood samples [19–21, 26, 27]. Therefore, extending the life of CTCs in patient blood samples for days will increase the availability of these rare cells, for use in real-time downstream analysis using next generation sequencing and/or proteomic analysis. Three cancer patient blood samples (breast, prostate and lung) were divided and incubated with or without SBTS for 6 days at RT. Blood samples were separated using ficoll, which has been previously described in isolating viable CTC from blood samples [41, 49–52]. The buffy coat layer was removed and incubated with CellTracker™ Blue CMAC cell viability stain as recommended by the manufacturer, then cells were centrifuged to remove free dye. Blue CMAC is a membrane permeable stain that is well retained by live cells and is fixable by formaldehyde. The cells were then filtered CellSieve™ microfilters, fixed and stained with the standard CTC marker antibodies (see Methods).

The prostate and lung cancer samples had no live CTCs as detected by our isolation procedure, whereas the breast cancer sample had 4 detectable live CTCs (CMAC Blue+, CK+, EpCAM+, CD45-) after 6 days in SBTS. The lack of CTCs isolated from the other patient samples can be attributed to a number of factors, including the fact that some cancer patients do not have detectable CTCs in their blood samples and that the ficoll separation step can lead to losses of CTCs [53–56].

**Fig. 3:**
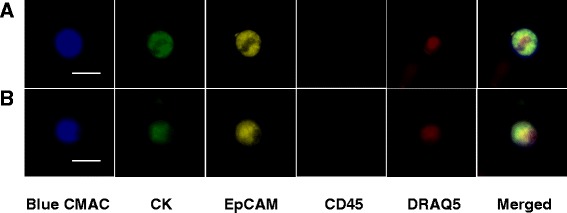
Representative images of CTCs isolated from breast cancer patient whole blood. Blood sample was kept at room temperature for 6 days and processed as described in the text. Rows **a** and **b** (*from left to right*) show images of captured CTCs stained for viability using Blue CMAC (*blue*), cytokeratin (CK) staining (*green*), EpCAM staining (*yellow*), Alexa Fluor 594 CD45 staining (*olive*), DNA staining (*red*) and merged figures, respectively. Bars indicate 20 μm

Figure 3, panels a and b show 2 live CTCs isolated from a breast cancer patient sample treated with SBTS and stored for 6 days at room temperature. The cells are alive and intact since Blue CMAC stain is retained within the cells. Cells are also positive for cytokeratin (green) and EpCAM (yellow), negative for CD45 and have an intact nucleus (red).

Duplicate patient blood samples treated with buffer and stored for 6 days at room temperature were also filtered and stained. Neither live nor dead CK+, EpCAM+ cells were found on the filters of blood samples stored without SBTS. The other 2 CTCs isolated from the breast cancer patient sample retained the Blue CMAC viability stain, but showed cell damage (not shown).

The data presented demonstrates that the SBTS preserves cell viability, even in harsh medium such as whole blood, which is detrimental to CTCs survival. Although the CTCs in the breast cancer patient sample were viable, we did not attempt their propagation in cell culture because culture conditions that allows for reproducible CTC growth without altering their phenotype has yet to be described. However, these initial experiments which show viability of CTCs suggest that when culture techniques for CTCs are developed, the SBTS is well suited for long term storage and transport of patient derived blood samples.

## Conclusions

A number of technologies have been developed to isolate live CTCs. However, since CTCs are rare and unstable in whole blood, isolation procedures must be performed rapidly after blood collection [19–21, 26, 27]. A six day time point for blood storage SBTS was chosen based on our results with cell culture (Table 1) and live MCF-7 cells spiked into normal whole blood (Figs. 1 and 2). Additionally, the 6 day period was chosen to represent an extreme case for transcontinental transportation of blood samples. The benefits of having a simple, low cost and non-toxic treatment of patient blood samples that preserves live CTC in blood samples for up to 6 days at ambient temperature will greatly enhance the ability to analyze CTCs, because clinicians and researchers will have more time to process these samples or ship them between research facilities. M. Ignatiadis et al. [57] succinctly wrote in their paper about this problem: “However, an important limitation of our study is that all patients were recruited in one center and the CTC analysis was done in one laboratory. An international, prospective, multicenter trial with different participating laboratories in which issues of stability during shipment of the samples, interlaboratory reproducibility of the multimarker assay, and validation of our results in a diverse patient population is urgently needed.”

## Abbreviations

CK: Cytokeratin
CMAC: 7-amino-4-chloromethylcoumarin
CTC: Circulating tumor cells
EpCAM: Epithelial cell adhesion molecule
FBS: Fetal bovine serum
SBTS: sugar based transportation solution
